# Geochemistry and provenance of Mesozoic sandstones in Khon Kaen Geopark: Implication for tectonics of the western Khorat Plateau of Thailand

**DOI:** 10.1371/journal.pone.0284974

**Published:** 2023-04-26

**Authors:** Vimoltip Singtuen, Burapha Phajuy, Apussorn Anumart, Punya Charusiri, Natnicha Chawthai, Heiner Heggemann

**Affiliations:** 1 Department of Geotechnology, Faculty of Technology, Khon Kaen University, Khon Kaen, Thailand; 2 Department of Geological Sciences, Faculty of Science, Chiang Mai University, Chiang Mai, Thailand; 3 Department of Mineral Resources, Ministry of Natural Resources and Environment, Bangkok, Thailand; 4 MESA CE, Faculty of Science, Chulalongkorn University, Bangkok, Thailand; 5 Hessisches Landesamt für Naturschutz, Umwelt und Geologie, Wiesbaden, Germany; Chinese Academy of Geological Sciences, CHINA

## Abstract

Khon Kaen Geopark, representing an area of dinosaur fossil diversity, was selected for investigations to reveal the origin and tectonic setting of the Khorat Group. The area occupied by Mesozoic sedimentary rocks of four formal formations of the Khorat Group, namely the Phra Wihan Formation (PWF), Sao Khua Formation (SKF), Phu Phan Formation (PPF), and Khok Kruat Formation (KKF). A field investigation and macroscopic observations suggested that the immature sedimentary rocks of the study area are mainly clast-supported, pebbly sandstone and siltstone with few calcretes. The 50 rock samples that were selected for petrographical and geochemical investigations revealed that the sandstones of the PWF and PPF are quartz arenite and sublitharenite, with some subarkose, whereas those of the SKF are mainly subarkose and sublitharenite. In addition, the KKF dominantly presents sublitharenite with pebbles and calcretes. Mesozoic sandstones contain quartz, feldspars, various types of rock fragments, and accessory minerals (biotite, muscovite, zircon, and tourmaline), with siliceous, ferrous, and calcareous cement. Petrographic (Q–F–L) and geochemical (major and trace element) data suggested that the sources of sediments are mostly quartzose sedimentary rocks and some felsic-intermediate igneous rocks. Chondrite-normalized rare earth element patterns indicated that the origins of the studied sandstones are quartzose sedimentary rocks deposited in a passive continental margin or an upper continental crust. Geochemical traits of the sedimentary successions demonstrated that the provenance of the Khorat Basin prior to reworking by fluvial processes was situated in the passive continental margin or recycled orogen of the paleo-volcanic arc during the Mesozoic period.

## Introduction

The Khon Kaen Geopark (KKGp) is one of the national geoparks of Thailand, located in the Khorat Plateau, a part of the Indochina Terrane. The area is situated in the Wiang Kao and Phu Wiang districts, Khon Kaen Province, and the western part is protected and conserved by the Phu Wiang National Park. The highest peak of the mountain range is at 844 m msl. The lowest level of the foothills is located at 210 m msl and the sedimentary basin is large, flat, and wavy/folded in the middle. Most of the area comprises red sedimentary rocks belonging to the Mesozoic Khorat Group, comprising the Phra Wihan Formation (PWF), Sao Khua Formation (SKF), Phu Phan Formation (PPF), and Khok Kruat Formation (KKF). The Khorat Group is a red-bedded sedimentary rock stratum, which formed within the continent during the Mesozoic era, consisting mainly of sandstone, siltstone, shale, and pebble sandstone associated with calcrete, rock salt, gypsum, and anhydrite. There are nine formations in the Khorat Group, namely the (i) Huai Hin Lat, (ii) Nam Phong, (iii) Phu Kradung, (iv) Phra Wihan, (v) Sao Khua, (vi) Phu Phan, (vii) Khok Kruat, (viii) Maha Sarakham, and (ix) Phu Thok formations.

**Fig 1:**
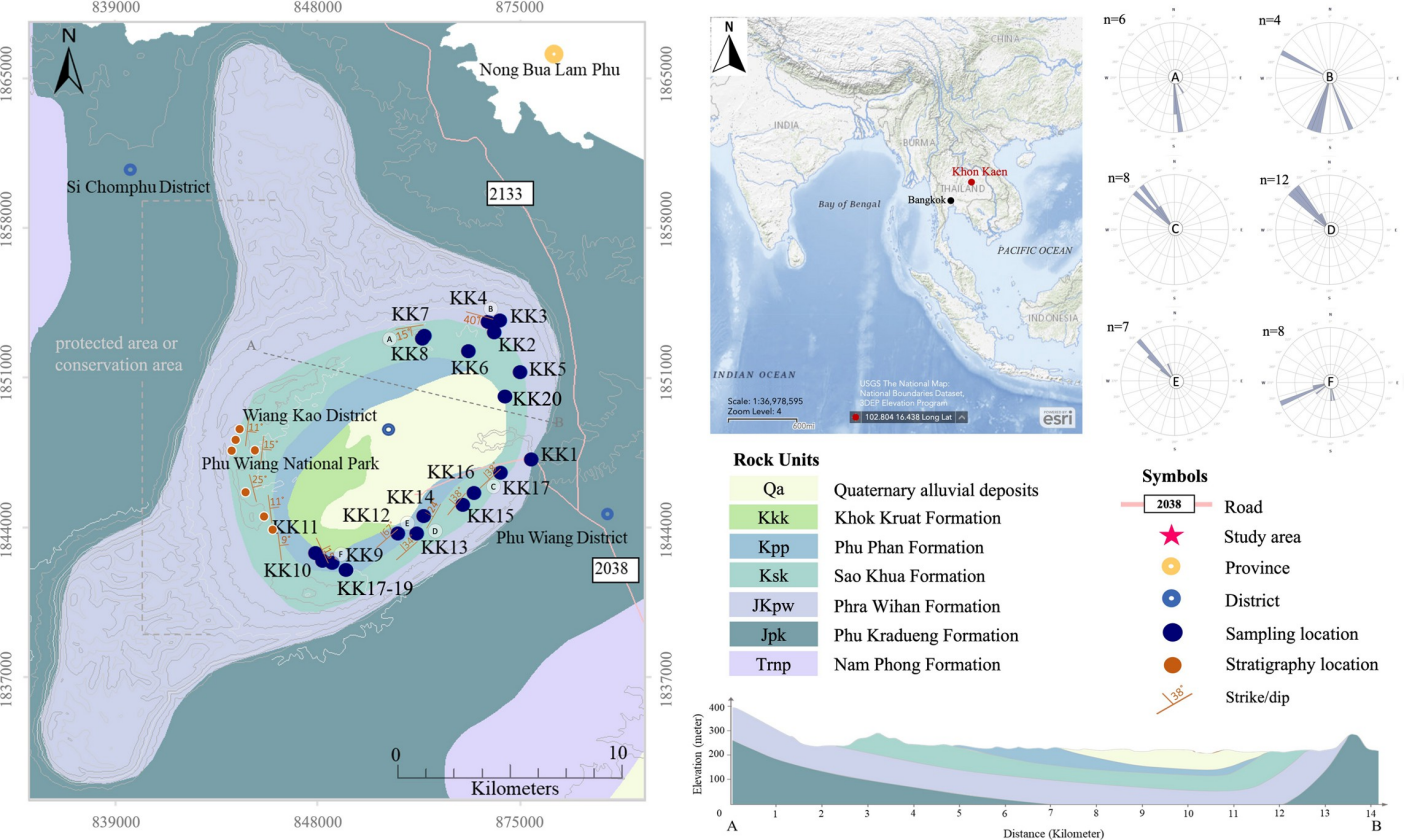
Geologic map of Khon Kaen Geopark area showing the location of rock sampling and bedding attitudes, and the geologic section along AB showing the synclinal structure of the Khorat Group rocks. The stereonet diagrams present the orientation of the bedding planes in each location (A‒F). The geological data is from [9].

Field observations indicate multiphase syncline geomorphology including high mountains and a valley in the region’s west (Fig 1). This may be owing to the formation process of collision between the mainland Indochina and Sibumasu Terranes (Indosinian orogeny) that took place during the late Triassic [1–4]. A series of uplift movements and tectonic faulting during this collision led to the formation of several inter-mountain thermal sag basins, which were filled with continental sediments during the Jurassic-Cretaceous (e.g., Khorat Group, South China Sea, Khorat Basin, Vietnam, Cambodia, and southern part of Laos) [5, 6].

U-Pb zircon dating was performed to determine the source of the Khorat Group sediment associated with the Qinling Belt in China and revealed that the age modes are 2456±4 Ma, 2001±4 Ma, 251±3 Ma, and 168±2 Ma [3]. Moreover, zircon fission tracks from sandstone on the west part of the Khorat Plateau were assessed to determine the maximum depositional ages of the Phu Kradung Formation, PWF, SKF, and KKF as 141±17 Ma to 210±24 Ma, 114±6 to 125±18 Ma, 160±6 Ma, and 133±13 Ma, respectively, suggesting that the sediment of the Khorat Plateau came from the Qinling Belt and the large basin parallel to the mountain range in the Lasa Plate and Eurasia Plate collision event during late Jurassic to early Cretaceous [5, 7, 8].

The KKGp was the first dinosaur fossil excavation area in Thailand [10]. In addition to dinosaur fossils, there are abundant vertebrate fauna fossils and non-marine bivalves in the Mesozoic sedimentary rocks of the SKF, spanning from the Jurassic to the middle Cretaceous [11–13]. This area presents a high diversity of dinosaur fossils, and five new dinosaur species were identified here. The first sauropod fossil of Thailand, *Phuwiangosaurus sirindhornae*, was discovered in the Jurassic SKF. The other four dinosaur species that were discovered for the first time in the KKGp were *Siamotyrannus isanensis*, *Kinnareemimus khonkaennsis*, *Phuwiangvenator yaemniyomi*, and *Siamosaurus suteethorni* [14–27].

There are many scholars studying stratigraphy and paleontology in the KKGp; however, the geochemical characteristics have not been systematically classified in terms of the petrographic name and provenance of sedimentary rocks. The Mesozoic Khorat Group within the KKGp, western Khorat Plateau can be divided into 4 formations and records the importance of local structures. Therefore, this research will be a necessary supplement to describe the geochemical characteristics and make an interesting addition to knowledge of the complicated Khorat Plateau region. The consideration of the region’s structural complexity and adequately researched provenance sources result in logical conclusions that can provide importance for geoscience and the earth’s evolution to the geopark.

## Geologic setting

**Fig 2:**
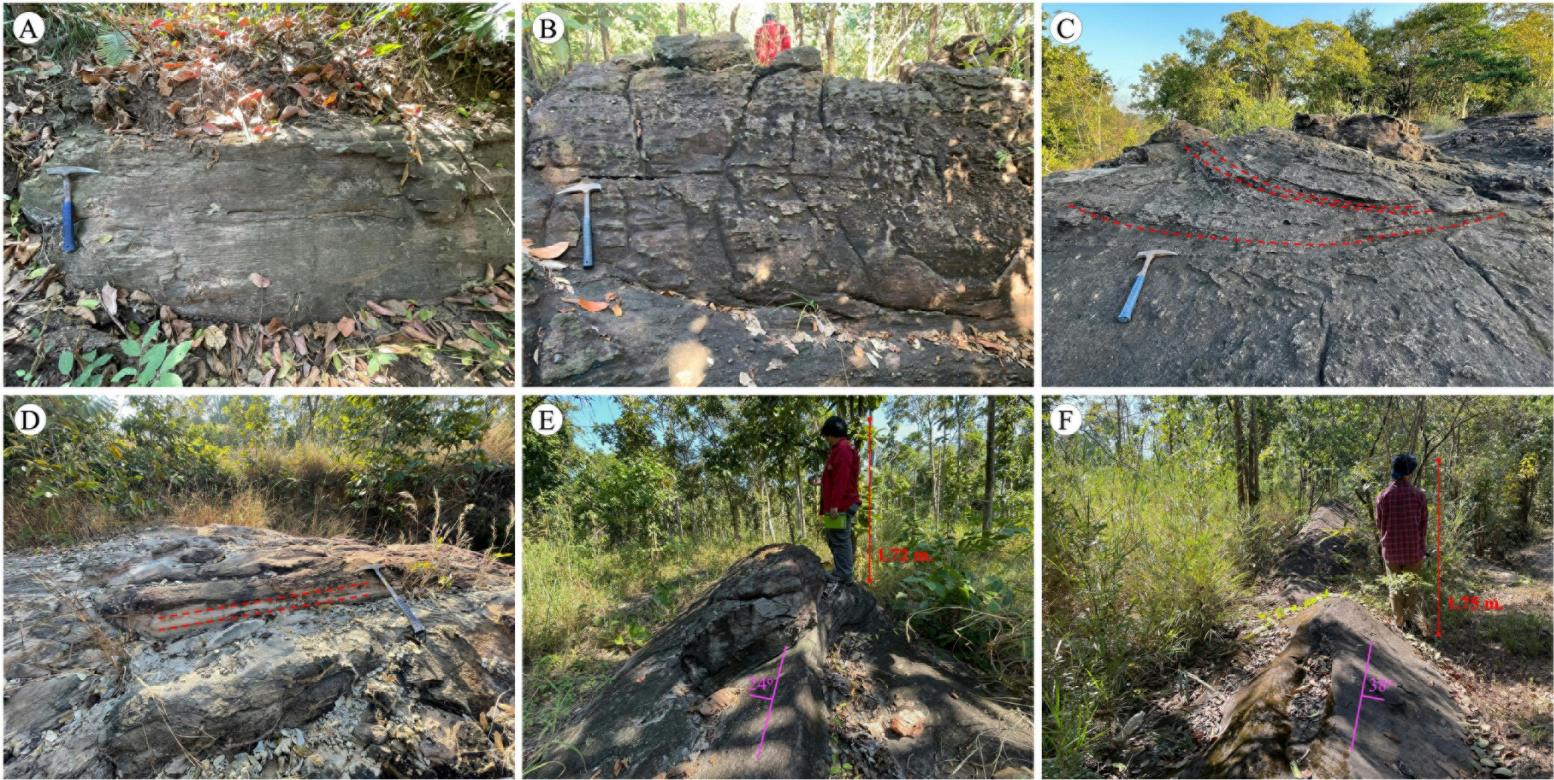
Outcrops of sedimentary rocks in Khon Kaen Geopark. (A) White sandstone of the Phra Wihan Formation at 48Q 216316E 1852542N, (B) maroon pebbly sandstone of the Sao Khua Formation at 48Q 212405E 1842716N, (C) pebbly sandstone and cross-bedding of the Phu Phan Formation at 48Q 208082E 1841546N, (D) white sandstone of the Phu Phan Formation at 48Q 211484E 1842718N, (E) maroon sandstone of the Sao Khua Formation at 48Q 214392E 1843976N (looking north), and (F) maroon sandstone of the Sao Khua Formation at 48Q 214911E 1844509N (looking south).

The KKGp has a unique topography, comprising a large plain bordered by hills and mountains. The park has only one entrance (Fig 2). There is a basin at the region’s center, with a rolling landscape enclosed by cliffs. The outermost mountain comprises two rings of resistant bedrock, with a steep slope and cuesta landform that reaches an elevation of 844 m. This area is dominated by non-marine rocks of the Khorat Group, which comprises the PWF, SKF, PPF, and KKF [9]. These sedimentary rocks—almost entirely red—are a red bed succession from the Mesozoic era, comprising mudstone, siltstone, sandstone, conglomerate, and freshwater limestone [11]. Abundant fossils are mainly found in the Sao Khua strata, whereas dinosaur footprints can be found in the Phra Wihan strata [28]. Furthermore, landforms, such as cliffs and waterfalls, are mainly observed in the Phra Wihan and Phu Phan strata.

White fine-to-coarse-grained sedimentary rocks of the PWF largely comprised of sandstones interbedded with siltstones, mudstones, and conglomerates (S1 Table), deposited in thin braided streams in the early Cretaceous (Barriasian to Barremian, 125–145 Ma) based on palynological data [10, 12, 29, 30]. In addition, based on research on *Sphenopteris goepperti*, it is suggested that cyclic sequences of reddish-brown sandy mudstone of the SKF were deposited on top of semi-arid meandering rivers interbedded with sandstone, siltstone, and conglomerate during the late Jurassic to early Cretaceous [23, 24, 30, 31]. This formation is among the richest fossil formations from the Khorat Group, comprising freshwater hypsodont sharks, actinopterygian fishes, turtles, crocodilians, and dinosaurs [16]. Through analysis of its vertebrate fossil content, the age of the SKF is dated to the early Cretaceous [17], whereas research on non-marine bivalves suggests that this formation was deposited during the late Barremian [24].

The light grey sandstone and conglomerate of the PPF appear to be poorly sorted medium-to-coarse-grained quartz, green volcanic rocks, and chert, with expansive planar and trough cross-bedding. This formation was deposited in braided streams in a humid to semi-arid environment during the early Cretaceous [12, 30]. Moreover, the reddish-brown stratum of the KKF (sandstones, siltstones, mudstones, and conglomerates, interbedded with gypsum lenses and calcrete nodules) was deposited in meandering rivers, in a semi-arid to an arid environment [30], during the early-middle Cretaceous (dated by freshwater bivalves [32]). The different lithologic and depositional aspects found in previous works [10, 12] are of interest to the current work for identifying and classifying the petrographical and geochemical characteristics of the Mesozoic sandstones in detail.

## Methodology

Initial field observations, samples, and measurements were taken in the Khon Kaen National Park (Fig 1) to determine the structure, orientation, etc. This area shows many geological structural features, including folds, faults, joints, and primary structures of sedimentary rocks. Fifty samples were collected from the early Cretaceous sedimentary rocks of the Khorat Group (PWF, SKF, PPF, and KKF) for study in petrographic and geochemical laboratories. Representative rocks were gathered to be studied in the petrographic lab for texture, mineral content, rock name identification (400-point counts, see S2 Table), and sedimentary characteristic classification. A petrographic examination was conducted on all 47 fresh rocks distributed among every rock unit in the studied areas. Moreover, at the Department of Geotechnology of Khon Kaen University, photomicrographs were taken using ZEN 3.4 (blue edition) core imaging software, using ZEISS (Carl Zeiss NTS Ltd., Oberkochen, Germany) imaging and Motic microscope BA310POL. Powder samples (200 μm) were made from finely chosen rock chips to detect chemical properties, individual rock names, and modification processes in the Department of Geological Sciences, Faculty of Science, Chiang Mai University.

For the geochemical study, 34 typical samples with the least amount of alteration, weathering, and replacement by secondary minerals (carefully evaluated under a polarizing microscope) were chosen for whole rock analysis using X-ray fluorescence (XRF76C) and inductively coupled plasma atomic emission spectroscopy (ICP95A). Analysis of the major oxides (SiO_2_, TiO_2_, Al_2_O_3_, Fe_total_, MnO, MgO, CaO, Na_2_O, K_2_O, and P_2_O_5_) was performed using the Axios model at SGS (Thailand) Co., Ltd (lower reporting limit: 0.01%; Bangkok, Thailand), see S3 Table. For low-atomic-number elements, lithium metaborate fusion was employed, whereas, for higher-atomic-number elements, the pressed pellet technique was used. Fusion is a technique for analyzing key elements, which involves melting the sample with flux and casting it into a glass disc. In addition, trace elements (Rb, Sr, Zr, Y, Nb, Ni, Cr, V, Sc, Hf, Th, and Ta) and rare earth elements (REEs; La, Ce, Pr, Nd, Sm, Eu, Gd, Tb, Dy, Ho, Er, Tm, and Yb) were analyzed using the 5300DV ICP-OES and nexion300X ICP-MS of SGS-CSTC Standards Technical Services Co., Ltd. (Beijing, China), see S4 Table.

## Results

### Field observation and petrography

The sampling location comprised 20 sites distributed in the KKGp, excluding the protected area of the Phu Wiang National Park (Table 1). Fig 2 shows representative pictures of outcrops of road cuts, in situ rock, and geomorphological sites (e.g., rock pillar, mountain, and cliff) with planar bedding and cross-bedding with some calcretes. The main structure comprises multi-phases of syncline and high mountains surrounding a valley. Based on field observation, the studied area was divided into six areas (A, B, C, D, E, and F) for analyzing structural geology, as shown in Fig 1.

**Table 1:** Sampling location and petrographic data of studied clastic sedimentary rocks in Khon Kaen Geopark.

sample	location	fm	hand spimecen	composition						grain size (mm)	Petrographic Name
				%clast	%matrix		%Q	%Ft	%Lt		
KK1-1	48Q 217490 1845994	PW	sandstone	94.09	5.09		91.48	4.12	4.40	0.22–0.56	quartz arenite
KK1-2		PW	sandstone	89.39	5.65		91.10	4.15	4.75	0.10–0.55	quartz arenite
KK1-3		PW	sandstone	89.64	5.52		90.94	4.16	4.90	0.13–1.19	quartz arenite
KK2-1	48Q 216041 1851981	PW	sandy mudrocks	54.13	8.26		53.60	19.60	26.80	0.02–0.06	lith arenite
KK2-2		PW	sandy mudrocks	51.99	7.49		53.78	18.49	27.73	0.02–0.05	lith arenite
KK3-1	48Q 216316 1852542	PW	calcrete	54.95	2.97		69.37	18.47	12.16	0.08–0.11	subarkose
KK3-2		PW	calcrete	62.53	10.74		57.04	23.47	19.49	0.01–0.11	subarkose
KK4-1	48Q 215776 1852503	PW	sandstone	95.82	1.82		83.02	7.17	9.81	0.13–0.75	sublitharenite
KK5-1	48Q 217122 1850109	SK	sandstone	94.83	2.87		91.18	4.48	4.33	0.56–1.35	quartz arenite
KK5-2		SK	sandstone	71.22	3.90		53.00	22.97	24.03	0.09–0.17	subarkose
KK5-3		SK	gravelly sandstone	93.35	4.19		92.74	4.39	2.87	0.62–1.01	quartz arenite
KK5-4	48Q 217159 1850045	SK	gravelly sandstone	88.51	6.22		91.00	4.64	4.35	0.12–1.14	quartz arenite
KK6-1	48Q 214864 1851142	SK	sandstone	87.82	7.46		76.27	12.37	11.36	0.13–0.57	subarkose
KK7-1	48Q 212955 1851936	SK	sandy mudrocks	65.85	6.10		54.18	24.30	21.51	0.02–0.11	subarkose
KK7-2		SK	sandy mudrocks	73.00	11.00		54.49	23.08	22.44	0.02–0.12	subarkose
KK8-1	48Q 212854 1851800	SK	sandstone	81.61	10.11		58.53	21.52	19.95	0.05–0.15	lithic arenite
KK9-1	48Q 208514 1841448	PP	sandy conglomerate								lithic arenite
KK9-3		PP	sandy conglomerate	91.13	7.30		91.42	4.56	4.01	0.31–4.81	lithic arenite
KK10-1	48Q 208082 1841546	PP	gravelly muddy sandstone								lithic arenite
KK10-2	48Q 208036 1841525	PP	gravelly sandstone	94.43	2.94		90.57	4.88	4.55	0.16–1.02	quartz arenite
KK10-3		PP	gravelly sandstone	96.34	1.34		90.87	4.94	4.18	0.84–1.46	quartz arenite
KK11-1	48Q 207786 1841913	PP	sandstone	94.92	3.98		91.73	4.19	4.08	0.36–0.90	quartz arenite
KK11-2		PP	sandstone	84.24	4.68		77.27	8.52	14.20	0.86–1.51	sublitharenite
KK12-1	48Q 211484 1842718	PP	sandstone	91.36	5.68		72.48	11.06	16.46	0.28–0.64	sublitharenite
KK12-2		PP	sandstone	87.84	4.96		62.89	14.16	22.95	0.20–0.64	sublitharenite
KK13-1	48Q 212322 1842712	SK	gravelly sandstone	87.80	8.29		61.39	14.72	23.89	0.50–6.25	sublitharenite
KK13-2		SK	sandstone	88.70	6.49		62.80	13.19	24.01	0.11–0.36	sublitharenite
KK13-3	48Q 212304 1842702	SK	subarkose	95.58		3.72	91.37	4.32	4.32	0.31–1.06	quartz arenite
KK13-4	48Q 212405 1842716	SK	gravelly sandstone	92.74		4.36	67.20	12.53	20.27	0.13–7.50	sublitharenite
KK14-1	48Q 212639 1843518	PP	sandstone	76.51		17.68	69.27	16.17	14.56	0.04–0.24	subarkose
KK15-1	48Q 214392 1843976	SK	sandstone	81.17		10.51	76.01	10.98	13.01	0.32–1.00	sublitharenite
KK15-2		SK	gravelly sandstone	82.47		5.17	72.92	9.38	17.71	0.50–2.66	sublitharenite
KK16-1	48Q 214911 1844509	SK	sandstone	86.86		10.71	65.87	10.58	23.54	0.19–0.80	sublitharenite
KK17-1	48Q 216132 1845425	SK	sandstone	95.79		2.75	91.49	4.51	4.01	0.31–1.77	quartz arenite
KK17-2	48Q 216120 1845416	SK	sandstone	91.26		3.64	68.82	6.45	24.73	0.11–0.52	sublitharenite
KK17-2*	48Q 206706 1843651	SK	sandstone								sublitharenite
KK18-1	48Q 206706 1843651	KK	pebbly sandstone	72.70		9.97	68.20	13.78	18.02	0.53–1.88	sublitharenite
KK18-2	48Q 208548 1841063	KK	pebbly sandstone	83.82		12.80	68.93	10.18	20.89	0.50–1.53	sublitharenite
KK18-3	48Q 208548 1841064	KK	pebbly sandstone								sublitharenite
KK18-5	48Q 208703 1841320	PP	pebbly sandstone								sublitharenite
KK19-1	48Q208627 1841068	PP	pebbly sandstone	88.15		7.65	69.00	10.24	20.75	0.50–1.25	sublitharenite
KK19-2		PP	pebbly sandstone	85.02		10.83	72.58	12.01	15.40	0.42–2.50	sublitharenite
KK20-1	48Q 217363 1849514	PP	sandstone	84.28		14.00	56.68	20.16	23.16	0.25–0.79	sublitharenite
KK20-2		PP	sandstone	84.60		11.98	53.55	20.77	25.68	0.23–0.81	sublitharenite
KK20-3		PP	sandstone	87.29		10.22	71.21	13.00	15.79	0.63–1.31	sublitharenite
KK20-4		PP	sandstone	67.32		16.34	62.12	13.13	24.75	0.02–0.07	sublitharenite
KK21	48Q 207786 1841913	PP	sandstone	94.92		3.98	91.73	4.19	4.08	0.36–0.90	sublitharenite

The sedimentary stratum in area A presents the orientation E-W/10–17° S, whereas the bedding of area B is approximately NW-SE and dips 40° in the SW direction. In addition, areas C, D, and E present the same strike in NE-SW and dip values are 24–67° NW. However, the strike of area F is NNW-SSE dipping 10–16° in the NE direction. Thus, based on the data on the orientation of the geological structures, the KKGp may be affected by folding structures.

The PWF is made up of white-colored, thick-bedded, poorly to well-sorted, fine-to-coarse sandstone, pebbly sandstone, and siltstone. Moreover, the SKF comprises maroon, moderate-to-well-sorted, very fine-to-very coarse sandstone interbedded with pebbly sandstone, and sandy siltstone. The uppermost part of the PWF is conformably overlaid by the SKF. In addition, the SKF can be divided into two parts: lower (medium-to-thick-bedded sandstone interbedded with siltstone) and upper (reddish siltstone, sandstone, limestone, conglomerate, calcrete layer, and fossils) parts.

**Fig 3:**
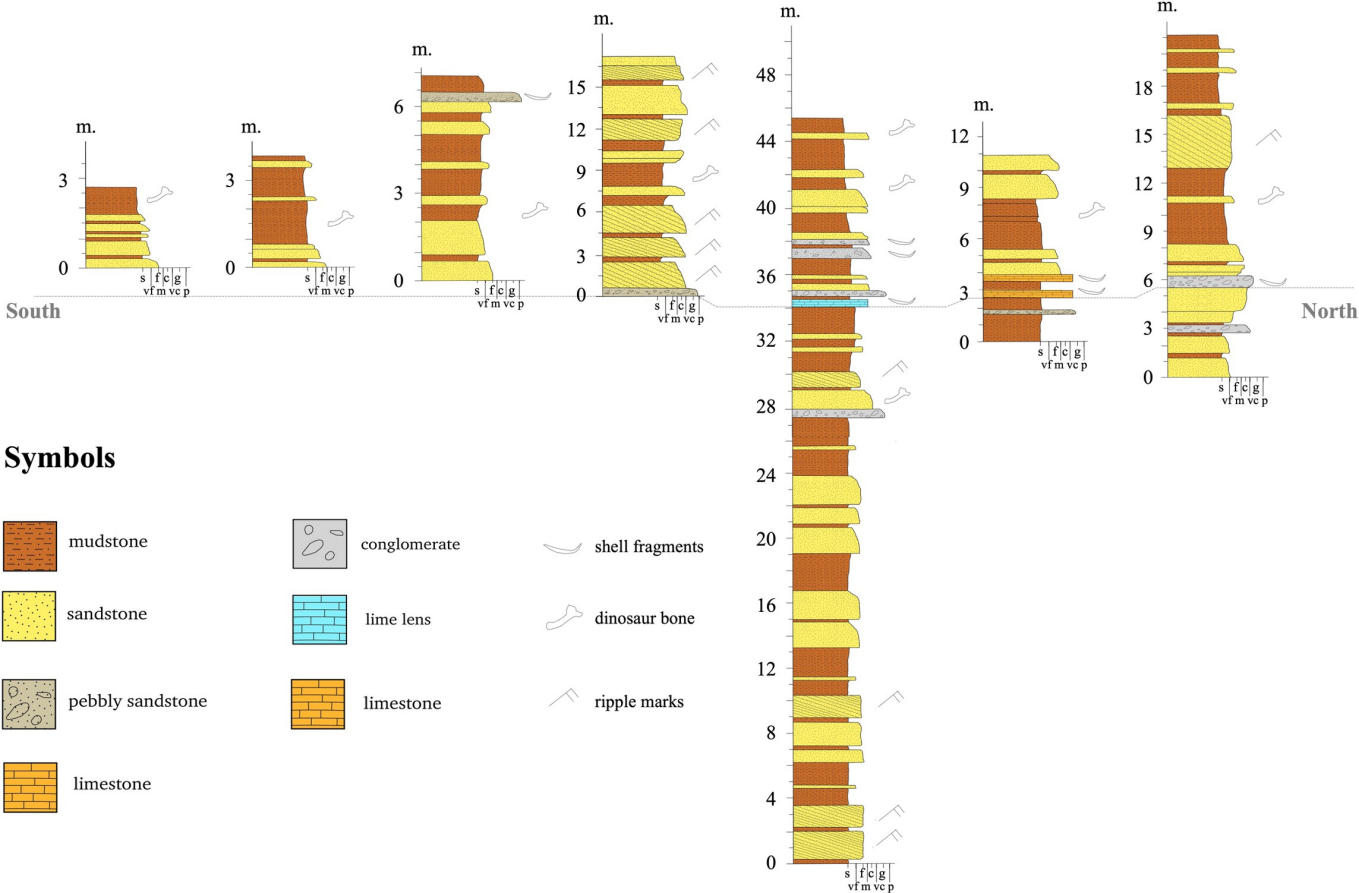
Stratigraphic columns in nine dinosaur excavation sites distributed in the Sao Khua Formation, Khon Kaen Geopark (stratigraphical and paleontological data from [28].

There are nine excavation sites in the SKF of the KKGp, presenting with four associated sedimentary facies, as shown in Fig 3: 1) channel fill deposit, 2) crevasse splay deposit, 3) lake and flood plain deposit, and 4) over bank deposit [28]. The maroon pebbly sandstone of the KKF is gravel-rich with coarse-to-very coarse, moderate-to-well-sorted sand.

**Fig 4:**
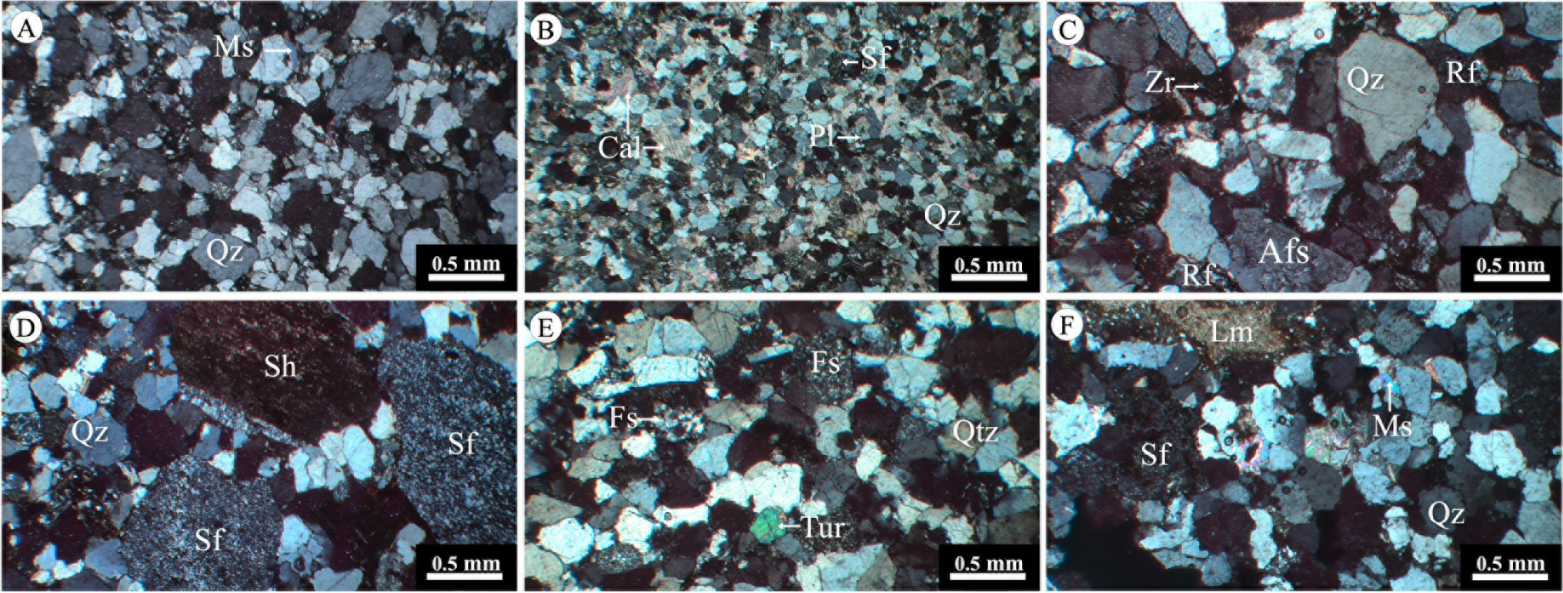
Photomicrographs in cross-polarized light of sedimentary rocks in Khon Kaen Geopark. (A) Quartz arenite of Phra Wihan Formation sample no. KK1-3, (B) subarkose of Phra Wihan Formation sample no. KK3-1, (C) sublitharenite of Sao Khua Formation sample no. KK15-1, (D) sublitharenite of Phu Phan Formation sample no. KK13-1, (E) sublitharenite of Sao Khua Formation sample no. KK20-1, (F) sublitharenite of Sao Khua Formation sample no. KK18-1. Qz: quartz, Pl: plagioclase, Afs: alkaline feldspars, Ms: muscovite, Tur: tourmaline, Zr: zircon, Cal: calcite, Sh: shale fragment, Sf: silicified rock fragments, Fs: felsic igneous rock fragments, Lm: limestone fragments.

Forty-seven lithological and petrographical studies conducted in 30 sites in the KKGp (Table 1) suggested that those rocks mainly comprise clast-supported, poor-to-well-sorted, subangular-to-angular shapes, and low-to-moderate sphericity clastic sedimentary rocks (Fig 4). The cementation is composed of three types: siliceous, ferrous (Fe-rich), and calcareous. These clastic sedimentary rocks comprise quartz, feldspars, plagioclase, tourmaline, muscovite, zircon, and opaque minerals. In addition, these rocks comprise various rock fragment types: shale/argillite (very fine-grained with lamination), quartzose sedimentary rocks (siltstone/sandstone), limestone (carbonate-rich), silicified rocks/quartzite (quartz-rich with sutured texture), and phyllite (very fine crystals with foliated texture), as well as felsic igneous rocks with some epidote and perthite.

**Fig 5:**
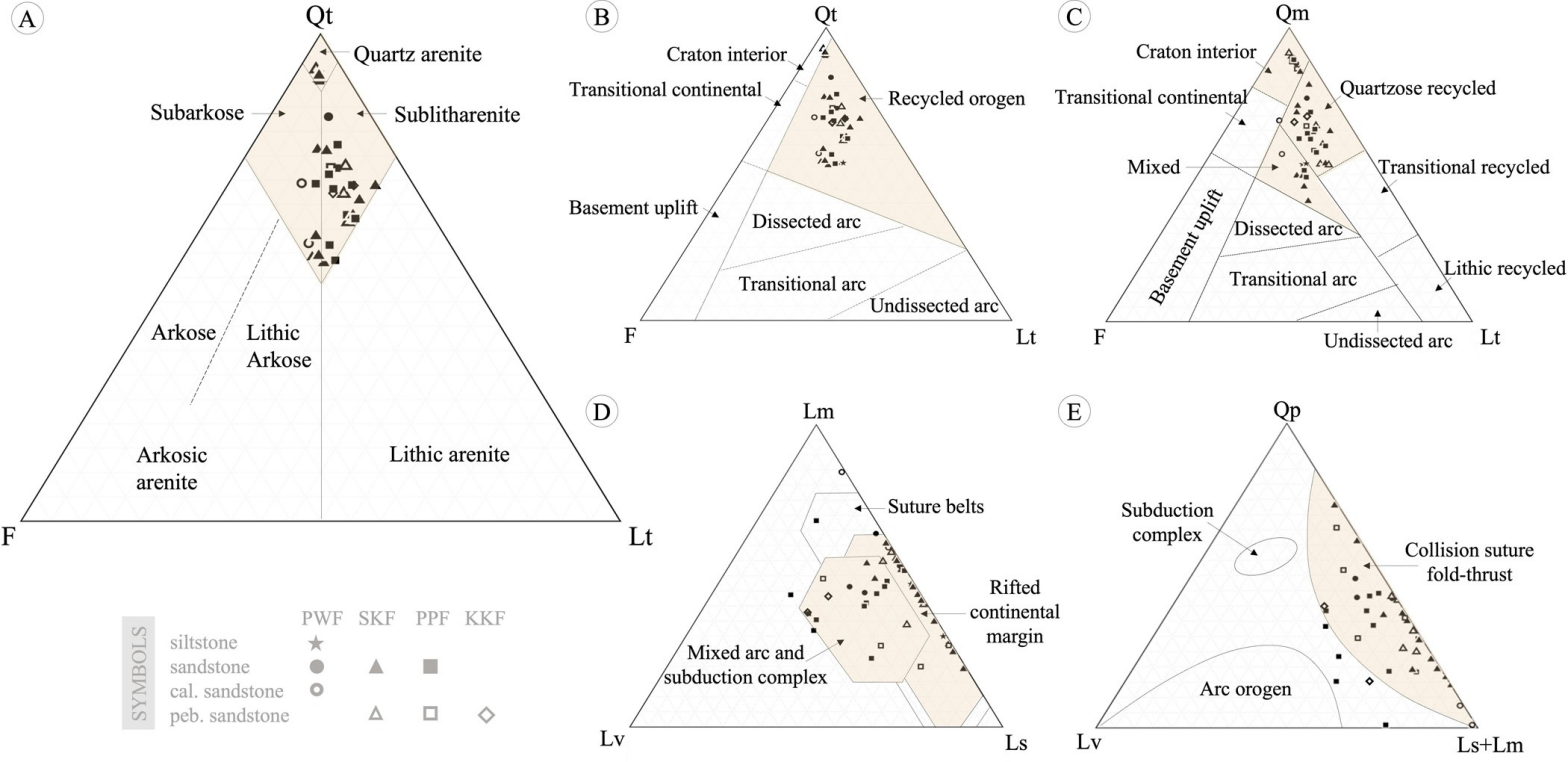
Diagrams of plotted petrographic data of sedimentary rocks in Khon Kaen Geopark. (A) Qt–F–Lt name classification diagram [34], (B) Qt–F–Lt, and (C) Qm–F–Lt diagrams with tectonic fields [35], (D) Lm–Lv–Ls diagrams with tectonic fields [36], and (E) Qp–Lv–(Ls+Lm) diagrams with tectonic fields [37]. Qt: total quartz, Qm: monocrystalline quartz, Qp: polycrystalline quartz, F: feldspar (plagioclase and K-feldspar), L: lithic fragment, Lt: lithic fragment and polycrystalline quartz, Ls: sedimentary lithic grains, Lv: volcanic lithic grains, Lm: metamorphic lithic grains.

Based on the quartz–feldspars–lithic fragments (Qt–F–Lt) classification [33, 34] in Fig 5A, the rocks from the PWF were classified as quartz arenite (Fig 4A), litharenite, and sublitharenite, with some subarkose (Fig 4B) (53.00%–69.37% quartz, 4.12%–23.47% feldspars, 4.33%–24.03% lithic fragments), whereas rock samples from the SKF were identified as sublitharenite (Fig 4C) and subarkose (53.55%–76.27% quartz, 9.38%–24.30% feldspars, 4.01%–24.73% lithic fragments). In addition, the petrographic data classified rocks from the PPF as quartz arenite, sublitharenite (Fig 4D and 4E), and some subarkose (61.39%–91.73% quartz, 4.19%–14.72% feldspars, 4.01%–20.75% lithic fragments). The rock samples from the KKF present only sublitharenite (68.20%–68.93% quartz, 10.18%–13.78% feldspars, 18.02%–20.89% lithic fragments) with pebbles and calcrets, as shown in Fig 4F.

The sandstones in the KKGp were plotted in the diagrams of feldspars–lithic fragments–quartz (Qt–F–Lt) and–monocrystalline quartz (Qm–F–Lt) with tectonic fields [35] mostly as recycled orogen with quartzose recycled, craton interior, and mixed sources (Fig 5B and 5C). In addition, the lithic fragments, metamorphic rocks (Lm)–volcanic rocks (Lv)–sedimentary rocks (Ls) diagram [36] shown in Fig 5D suggests that these rocks are related to rifted continental margin, mixed arc, and subduction complex. Moreover, these rocks were plotted in the diagrams of polycrystalline quartz (Qp) and lithic fragments [37] as collision suture fold-thrust (Fig 5E).

### Geochemistry

**Fig 6:**
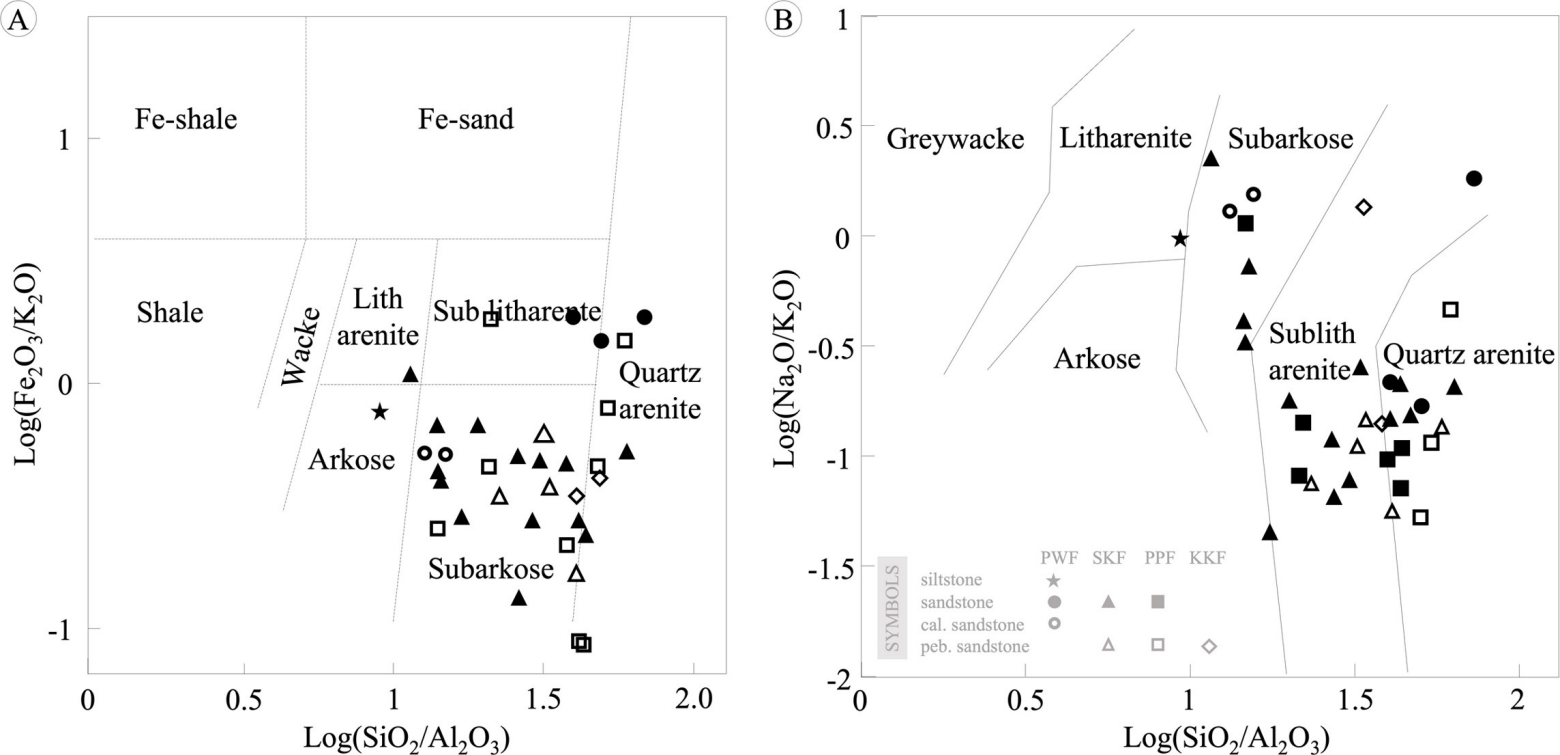
Chemical classification of sedimentary rocks in Khon Kaen Geopark. (A) log(Fe_2_O_3_/K_2_O) and log(SiO_2_/Al_2_O_3_) fields [38], and (B) log(Na_2_O/K_2_O) and log(SiO_2_/Al_2_O_3_) fields after [34].

Thirty-four representative samples were analyzed for major oxides, trace elements, and REEs (Table 2). The rock sequence of the PWF and SKF showed a wide rank of silica (SiO_2_ 62.12–97.55 wt% and 69.02–96.12 wt%, respectively), whereas the PPF and KKF presented 87.63–96.95 wt% and 84.41–95.25 wt%, respectively. The maroon sedimentary rocks presented Fe_2_O_3_ higher than 1%; however, the iron concentrations (Fe_2_O_3_) of white sandstone were lower than 1%. According to the relationship of Fe_2_O_3_/K_2_O and SiO_2_/Al_2_O_3_ [38] in Fig 6A, the PWF was classified as quartz arenite and subarkose, with a small amount of arkose, whereas rock samples from the SKF were identified as arkose and subarkose. The sandstones of the PPF were identified as quartz arenite, sublitharenite, and subarkose. However, the rock samples from the KKF were plotted in the subarkose and quartz arenite fields.

**Table 2:** Whole-rocks analysis for major oxides, trace elements, and REEs of studied clastic sedimentary rocks in Khon Kaen Geopark.

**Analysis no.**	Phra Wihan Formation																						Sao Khua Formation					
	KK1-2	KK1-3		KK-2-2		KK-3-1		KK3-2		KK-4-1		KK-5-1		KK-5-2			KK5-4			KK-6-1			KK7-2			KK-8-1		
**Major Oxides (wt%)**																												
SiO_2_	95.71	94.6		66.03		67.49		62.12		97.55		95.98		77.64			95.91			87.29			69.02			87.48		
TiO_2_	0.12	0.2		0.55		0.26		0.33		0.03		0.05		0.24			0.06			0.46			0.57			0.44		
Al_2_O_3_	1.91	2.29		7.1		4.32		4.7		1.33		2.21		5.25			1.63			5.82			5.89			5.96		
Fe_2_O_3_t	0.9	1.21		2.36		0.78		1.02		0.32		0.22		0.88			0.68			1.47			1.81			1.83		
MnO	<0.01	<0.01		0.08		0.2		0.26		<0.01		0.02		0.07			<0.01			0.16			0.14			0.12		
MgO	0.04	0.05		0.98		0.34		0.49		0.04		0.06		0.32			0.08			0.42			0.56			0.51		
CaO	0.05	<0.01		10.35		13.58		16		<0.01		0.01		7.1			<0.01			0.12			10.44			0.08		
Na_2_O	0.03	0.04		0.88		0.68		0.74		0.09		0.05		0.21			0.02			0.7			1.11			0.32		
K_2_O	0.18	0.19		0.9		0.44		0.57		0.05		0.24		0.67			0.15			0.99			0.51			0.81		
P_2_O_5_	0.06	0.03		0.11		0.06		0.07		0.02		<0.01		0.05			0.06			0.04			0.1			0.02		
**Trace Elements REEs (ppm)**																												
La	9.1	11.8		30.4		19.4		26.9		6.8		7.3		21.6			10.6			78.7			31.9			32.1		
Pr	2.1	2.92		7.49		5.59		6.47		1.76		1.58		5.84			2.49			15.84			8.27			8.38		
Nd	7.5	10.3		28.8		21.2		25		6		5.5		23.4			9.7			58.6			32.7			32.6		
Eu	0.23	0.35		1.11		0.93		1.17		0.17		0.16		0.97			0.52			2.31			1.78			1.28		
Gd	1.08	1.54		4.76		3.94		4.73		0.72		0.69		3.81			1.79			9.28			5.54			5.48		
Tb	0.19	0.25		0.81		0.67		0.83		0.09		0.09		0.62			0.27			1.48			0.86			0.9		
Dy	1.1	1.29		4.43		3.57		4.57		0.45		0.47		3.15			1.34			7.81			4.76			4.8		
Ho	0.22	0.24		0.85		0.65		0.86		0.08		0.09		0.56			0.24			1.4			0.92			0.9		
Er	0.67	0.72		2.56		1.85		2.34		0.25		0.26		1.56			0.63			3.89			2.88			2.56		
Tm	0.1	0.11		0.4		0.26		0.34		<0.05		<0.05		0.22			0.08			0.55			0.43			0.37		
Yb	0.6	0.7		2.5		1.6		2		0.2		0.3		1.3			0.5			3.1			2.8			2.2		
Ce	23.9	32.4		76.6		55.2		70.1		21.4		30.3		55.5			24.2			90.5			78.9			73.3		
Sm	1.3	2.1		6.3		4.2		5.7		1		1		5.1			1.9			10.5			6.4			6.3		
Th	3.1	4.2		10		5.7		6.3		1.3		2.5		5.1			2.2			4.9			9.1			8.2		
Ta	<0.5	<0.5		1.1		0.5		0.6		<0.5		<0.5		2			<0.5			0.7			1.1			0.7		
Nb	1	2		13		6		7		<1		<1		9			<1			7			12			8		
Zr	66.2	160		336		201		268		30.2		32.5		122			30.7			252			618			265		
Hf	2	5		9		5		7		<1		1		3			1			8			17			7		
Y	5.3	9.7		24.2		18.9		24.5		2.5		4.4		20.9			7.8			45.3			27.2			26.9		
Rb	10.6	9.9		48.2		22.7		25.7		2.9		11.1		29.3			8.3			37.4			24.1			40		
Sr	8.8	14.9		52.2		50.5		69.2		4.4		13.1		37.2			14.5			43.9			88.4			21.5		
Ga	3	3		10		5		5		1		2		5			2			7			6			8		
Lu	0.06	0.09		0.37		0.23		0.29		<0.05		<0.05		0.17			<0.05			0.46			0.42			0.32		
U	0.95	1.08		2.21		1.62		1.9		0.48		0.86		1.4			0.68			1.9			2.37			1.72		
Rb/Sr	1.205	0.664		0.923		0.450		0.371		0.659		0.847		0.788			0.572			0.852			0.273			1.205		
Th/U	3.263	3.889		4.525		3.519		3.316		2.708		2.907		3.643			3.235			2.579			3.840			3.263		
**Analysis no.**			Phu Phan Formation																									
			KK9-1		KK10-1		KK10-2		KK11-1		KK11-2		KK12-1		KK12-2			KK13-2			KK13-3			KK13-4			KK14	
**Major Oxides (wt%)**																												
SiO_2_			96.25		95.83		96.44		95.96		95.42		91.78		89.69			92.96			96.95			89.75			87.63	
TiO_2_			0.06		0.24		0.13		0.02		0.04		0.2		0.22			0.17			0.06			0.16			0.2	
Al_2_O_3_			1.55		1.76		1.91		2.21		2.4		4.2		4.06			3.41			1.53			3.88			6.03	
Fe_2_O_3_t			0.68		0.71		0.3		0.17		0.38		0.78		2.76			1.01			0.36			0.8			0.94	
MnO			0.03		<0.01		<0.01		0.02		<0.01		<0.01		0.03			<0.01			<0.01			<0.01			0.02	
MgO			0.07		0.07		0.07		0.08		0.06		0.35		0.33			0.14			0.06			0.21			0.56	
CaO			<0.01		<0.01		<0.01		<0.01		<0.01		0.09		0.07			0.02			<0.01			0.02			0.19	
Na_2_O			0.06		0.03		<0.01		0.04		0.05		0.04		0.06			0.07			0.04			0.05			1.23	
K_2_O			0.13		0.26		0.19		0.57		0.51		0.5		0.43			0.6			0.2			0.67			1.07	
P_2_O_5_			0.02		0.02		0.02		0.01		0.09		<0.01		0.01			0.16			0.04			0.02			0.02	
**Trace Elements REEs (ppm)**																												
La			15.9		13.4		8.1		8.6		9.4		11.3		18.4			12			10.6			15.6			9.7	
Pr			2.67		3.34		2.07		1.73		2.01		3.76		3.16			2.29			1.95			2.74			1.97	
Nd			9.7		10.9		7.6		6.5		7.6		13		11.5			8.2			7.2			9.4			7.2	
Eu			0.35		0.28		0.27		0.28		0.36		0.45		0.52			0.35			0.29			0.32			0.38	
Gd			1.47		1.4		1.04		1.1		1.45		1.58		1.95			1.33			1.1			1.37			1.13	
Tb			0.22		0.2		0.15		0.17		0.25		0.19		0.29			0.2			0.17			0.21			0.19	
Dy			1.19		1.09		0.76		1.05		1.4		0.9		1.6			1.05			0.93			1.08			1.09	
Ho			0.2		0.22		0.14		0.2		0.26		0.16		0.3			0.19			0.17			0.2			0.22	
Er			0.62		0.71		0.41		0.59		0.77		0.49		0.9			0.57			0.53			0.63			0.7	
Tm			0.14		0.11		0.06		0.09		0.11		0.07		0.14			0.08			0.07			0.1			0.12	
Yb			0.7		0.7		0.4		0.6		0.7		0.5		0.8			0.5			0.5			0.6			0.7	
Ce			32.6		38.9		20.8		18.5		21.1		37.2		85.9			25.1			20			28.3			20.8	
Sm			1.7		1.8		1.3		1.3		1.6		2.2		2.2			1.6			1.4			1.8			1.5	
Th			3.1		4.4		2.2		2.4		3.1		3.1		3.2			3.6			2.8			4.4			3.2	
Ta			<0.5		<0.5		<0.5		<0.5		<0.5		<0.5		<0.5			<0.5			<0.5			<0.5			<0.5	
Nb			<1		1		1		<1		1		3		4			4			<1			3			4	
Zr			41.7		156		52.7		33.1		73.3		51.5		91.2			56.4			40.6			88			106	
Hf			1		4		2		1		2		2		3			2			1			2			3	
Y			5.1		8.2		4.5		10.2		9.2		6.4		11.7			7			6.3			7.6			9	
Rb			5.9		11.3		9		23		24.4		17.3		17.7			33.2			10			34.9			45.2	
Sr			18.4		15		13.2		19.7		17.1		27.3		24.9			24.9			14.4			21.1			68.4	
Ga			2		4		2		2		3		4		5			3			3			4			7	
Lu			<0.05		0.08		<0.05		0.05		0.07		<0.05		0.09			<0.05			<0.05			0.06			0.08	
U			0.71		1.38		0.76		0.8		0.93		0.55		0.91			1.93			0.88			1.25			0.9	
Rb/Sr			0.321		0.753		0.682		1.168		1.427		0.634		0.711			1.333			0.694			1.654			0.661	
Th/U			4.366		3.188		2.895		3.000		3.333		5.636		3.516			1.865			3.182			3.520			3.556	
**Analysis no.**		Sao Khua Formation														Khok Kruat Formations						Sao Khua Formation						
		KK15-1		KK15-2		KK16-1		KK17-1		KK17-2		KK17-2*				KK18-1			KK18-3			KK19-2			KK20-1			KK21
**Major Oxides (wt%)**																												
SiO_2_		88.83		93.56		93.85		96.8		94.24		90.26				84.41			95.25			95.23			94.75			96.12
TiO_2_		0.21		0.08		0.12		0.04		0.06		0.26				0.11			0.06			0.11			0.2			0.03
Al_2_O_3_		4.42		2.87		3.06		2.06		3.45		5.14				2.48			2.37			2.31			2.88			2.16
Fe_2_O_3_t		1.06		0.59		0.36		0.16		0.35		0.86				0.63			0.44			0.4			0.39			0.16
MnO		<0.01		0.05		0.02		<0.01		0.03		0.03				0.09			0.06			0.02			<0.01			<0.01
MgO		0.19		0.11		0.14		0.05		0.11		0.27				0.12			0.13			0.11			0.09			0.05
CaO		<0.01		<0.01		<0.01		<0.01		<0.01		<0.01				6.86			0.1			<0.01			0.23			<0.01
Na_2_O		0.08		0.03		0.03		0.03		0.05		0.04				0.07			0.04			0.04			0.06			0.06
K_2_O		0.46		0.27		0.4		0.2		0.79		0.9				0.48			0.28			0.71			0.24			0.55
P_2_O_5_		0.02		0.01		0.02		<0.01		0.02		0.04				0.03			0.05			0.01			0.02			0.01
**Trace Elements REEs (ppm)**																												
La		20		14		14.9		8.7		12.3		22.8				10.9			9.6			12.9			16.4			8.5
Pr		4.27		4		2.98		1.76		2.12		4.04				2.27			2.66			2.79			2.44			1.64
Nd		15.3		14		11.1		6		7.5		14.3				8.6			10.5			10.2			8.5			6.3
Eu		0.6		0.46		0.5		0.17		0.35		0.54				0.43			0.57			0.35			0.3			0.28
Gd		2.37		1.72		1.93		0.8		1.09		2.34				1.58			2.04			1.43			1.3			1.15
Tb		0.33		0.23		0.36		0.12		0.18		0.37				0.26			0.35			0.18			0.19			0.2
Dy		1.5		1.18		2.02		0.59		0.87		2.13				1.39			1.74			0.96			0.93			1.07
Ho		0.25		0.2		0.39		0.11		0.15		0.43				0.26			0.32			0.17			0.17			0.2
Er		0.71		0.6		1.21		0.38		0.47		1.35				0.79			0.95			0.53			0.54			0.61
Tm		0.1		0.08		0.18		0.05		0.07		0.2				0.12			0.14			0.07			0.08			0.09
Yb		0.6		0.5		1.1		0.3		0.5		1.2				0.7			0.9			0.5			0.5			0.6
Ce		74		38.6		29.4		27		24.7		39.5				22.1			26.9			29.4			26.1			17.4
Sm		3.3		2.5		2.4		1		1.6		2.6				1.6			2.3			2			1.6			1.4
Th		4.8		4.5		2.9		2.7		3.1		6.3				3.3			3.2			3.6			3.3			2.3
Ta		<0.5		<0.5		<0.5		<0.5		<0.5		<0.5				<0.5			<0.5			<0.5			<0.5			<0.5
Nb		5		3		2		<1		2		5				4			3			1			3			1
Zr		88.7		57.7		44.2		35.6		87		152				66.7			71.5			98.6			69.5			30.7
Hf		3		2		1		1		3		4				2			2			3			2			1
Y		7.6		3.7		16.6		3.8		7.6		17.9				8.6			9			8.2			9.2			7.7
Rb		26.8		17.3		20		9.2		32.8		46.9				19.5			16.1			28.7			10.4			23.6
Sr		20.8		15.3		19.6		15.4		26.7		27.2				17			10.2			27.8			29.2			21.7
Ga		5		4		3		2		3		6				2			2			2			3			2
Lu		0.06		<0.05		0.13		<0.05		<0.05		0.16				0.08			0.09			<0.05			<0.05			<0.05
U		0.92		0.78		0.85		0.82		0.88		1.55				0.85			1.01			0.82			0.82			0.73
Rb/Sr		1.288		1.131		1.020		0.597		1.228		1.724				1.147			1.578			1.032			0.356			1.088
Th/U		5.217		5.769		3.412		3.293		3.523		4.065				3.882			3.168			4.390			4.024			3.151

Based on the diagram of log(Na_2_O/K_2_O) versus log(SiO_2_/Al_2_O_3_) [34], the PPF sandstone was plotted in the quartz arenite, lithic arenite, subarkose, and sublitharenite fields (Fig 6B). In addition, the SKF sandstone was classified as subarkose and sublitharenite, the PPF as quartz arenite and sublitharenite, and the KKF as sublitharenite. According to the isotopic systems of zircon-specific and whole-rock geochemistry of granitoids in Sukhothai Terrane and the western part of Indochina [39, 40], the geochemical characteristics are relatively consistent with the lithic arenite and sublitharenite in KKGp. However, SiO_2_ has been increased, while Al_2_O_3_ has been lost during feldspars and other inconsistent minerals weathering and alteration. The ratios of Th/U in PWF, SKF, PPF, and KKF are 2.579–4.525, 3.293–5.769, and 3.168–3.882, respectively that lower than Th:U of comparison granitoid (8.0–11.8) indicating that both Pb and Th have been lost from the zircon during subsequent tectonic events and sedimentary process.

### Provenance

**Fig 7:**
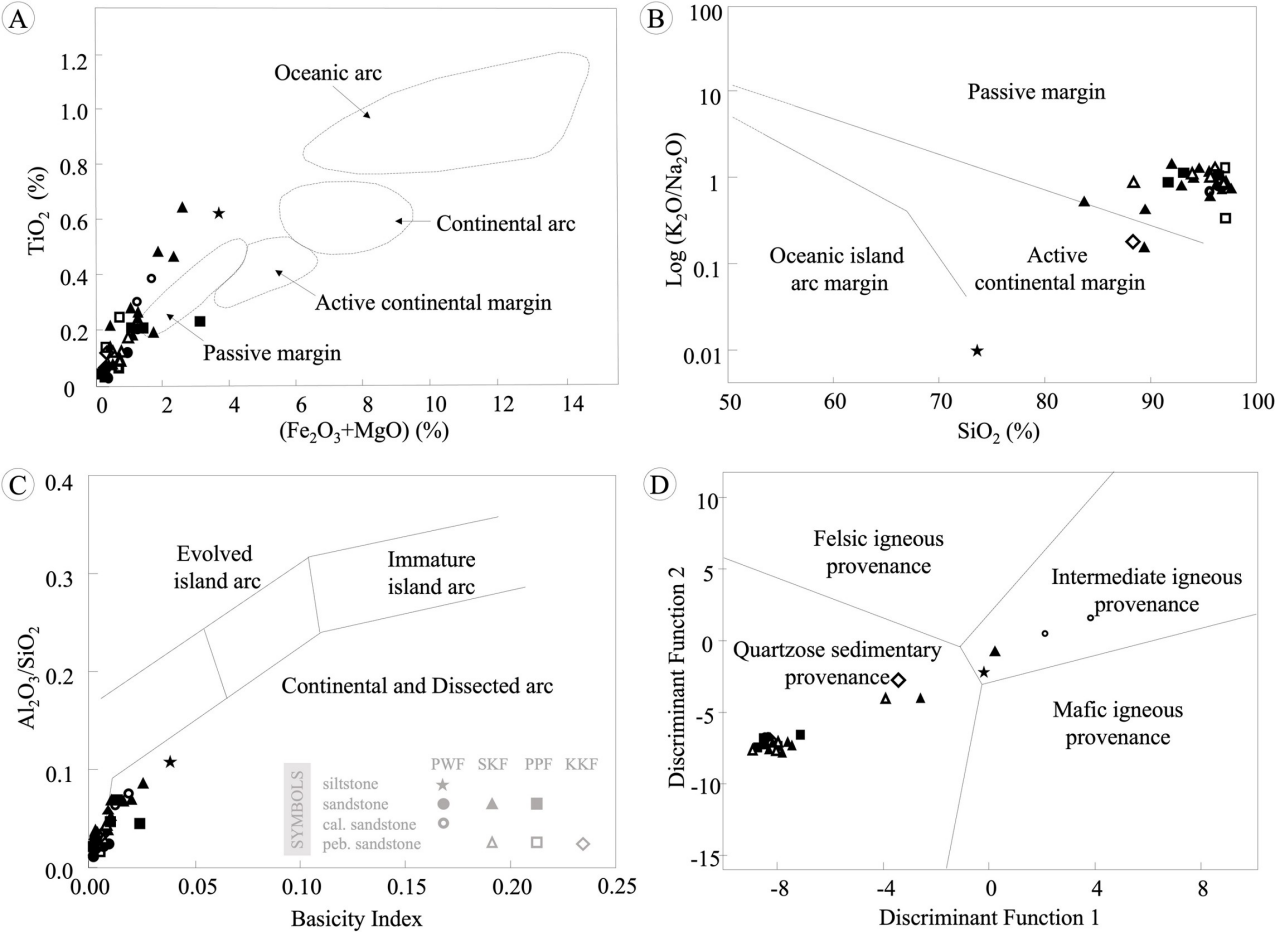
Tectonic-setting discrimination diagrams for the sedimentary rocks in Khon Kaen Geopark. (A) FeO+MgO vs. TiO_2_ fields [41], (B) SiO_2_–Log(K_2_O/Na_2_O) diagram [43], (C) basicity diagram [44, 45], and (D) discriminant function analysis using major oxides [42].

The relationships of major oxides (TiO_2_, Fe_2_O_3_, MgO, K_2_O, Na_2_O, SiO_2_, and Al_2_O_3_) can be used to classify the tectonic settings of sedimentary rocks [41–45]. The sandstones of the KKGp were mostly plotted in the passive margin field, whereas some fell in the active continental arc (Fig 7A–7C). The basicity index can be calculated by Eq 1.


$$\;\text{Basicity}\;\text{Index}\;=(\text{FeO}+\text{MgO})/\left({\text{SiO}}_{2}+K_{2}\text{O}+{\text{Na}}_{2}\text{O}\right)$$ (1)

Moreover, the following functions were used for classifying the provenance of sandstone using major oxides for Fig 7D.


$$DF1={-}_{1.773}{\text{TiO}}_{2}{+}_{0.607}{\text{Al}}_{2}{\text{O}}_{3}{+}_{0.76}{\text{Fe}}_{2}{\text{O}}_{3}{-}_{1.5}\text{MgO}{+}_{0.616}\text{CaO}{+}_{0.509}{\text{Na}}_{2}\text{O}{-}_{1.224}{\text{K}}_{2}\text{O}{-}_{9.09}$$ (2)

$$DF2{=}_{0.445}{\text{TiO}}_{2}{+}_{0.07}{\text{Al}}_{2}{\text{O}}_{3}{-}_{0.25}{\text{Fe}}_{2}{\text{O}}_{3}{-}_{1.142}\text{MgO}{+}_{0.438}\text{CaO}{+}_{1.475}{\text{Na}}_{2}\text{O}{+}_{1.426}{\text{K}}_{2}\text{O}{-}_{6.9}$$ (3)

**Fig 8:**
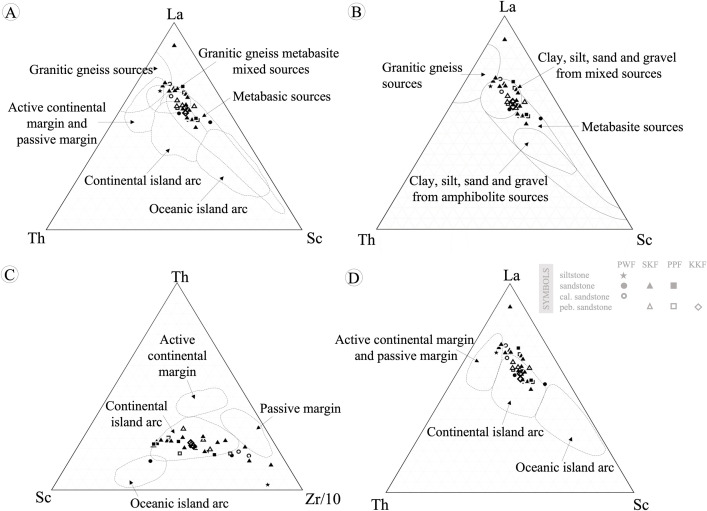
Ternary trace elements classification of sedimentary rocks in Khon Kaen Geopark. (A) La-Th-Sc discrimination diagram [45], (B) La-Th-Sc discrimination diagram [44], (C) Th-Sc-(Zr/10) discrimination diagram [46], and (D) La-Th-Sc discrimination diagram [47].

Ternary trace elements classification diagrams were used to classify provenance and tectonic settings. The La-Th-Sc discrimination diagrams [46] in Fig 8A and 8B suggest that the studied sandstones in the KKGp were from mixed sources (granitic gneiss metabasite, clay, silt, sand, and gravel). In addition, based on the Th-Sc-(Zr/10) discrimination diagram [41] and La-Th-Sc discrimination diagram [41] in Fig 8C and 8D, these sources were related to the continental arc setting. Thus, the PWF, SKF, PPF, and KKF of the Khorat Group may be formed from sediments of mixed sources (felsic-intermediate rocks) of the continental arc.

**Fig 9:**
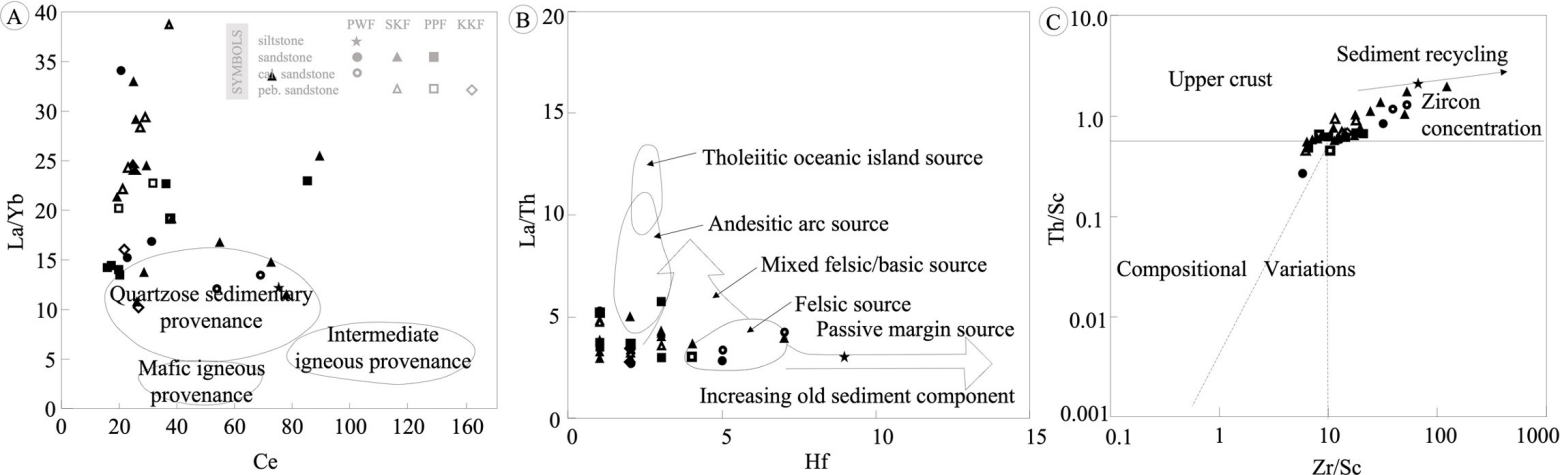
Chemical classification to determine source rock compositions of sedimentary rocks in Khon Kaen Geopark. (A) La/Yb versus Ce diagram [47], (B) La/Th versus Hf diagram [48], and (C) Th/Sc versus Zr/Sc diagram [49].

Based on the discriminant function analyses using major oxides [42], the sandstones were mostly classified as quartzose sedimentary (Fig 7D). Furthermore, some very fine-grained sandstones of SKF, siltstones of PWF, and calcareous sandstones of PWF were plotted in the intermediate igneous provenance field because these rocks have high CaO (wt%) from soluble cement, which affects the CaO value in the discriminant functions. However, similarities appeared when comparing REEs and immobile trace elements in the La/Yb versus Ce diagram [48], suggesting that the sandstones of the KKGp were formed from quartzose sedimentary provenances (Fig 9A). In addition, the La/Th versus Hf classification diagram [49] suggests that the sandstones of the KKGp were formed from the intermediate arc and felsic passive margin sources (Fig 9B). Furthermore, the Th/Sc versus Zr/Sc diagram [50] indicates that the sandstones are related to the upper crust and sediment recycling (Fig 9C).

### Comparison with previous studies

**Fig 10:**
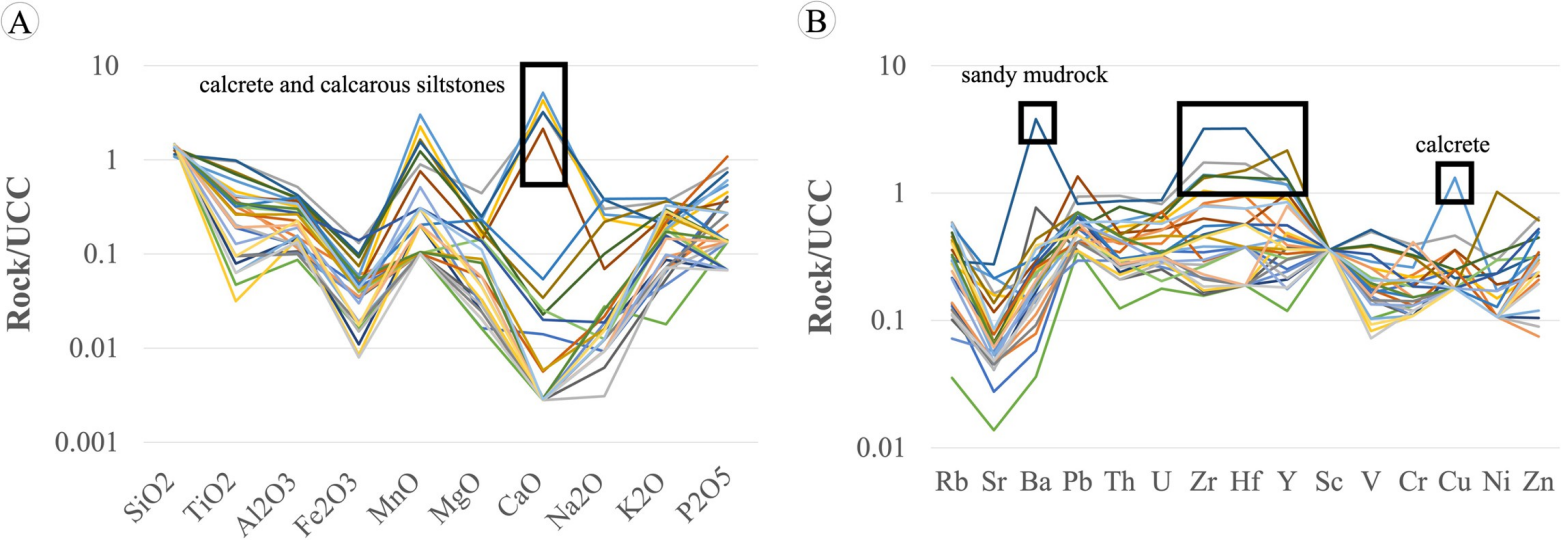
Upper continental crust (UCC)-normalized patterns of the KKGp sedimentary rock. (A) Major-minor oxides and (B) trace elements. Data for UCC are from [51].

The major and minor oxides normalized analyses were compared with the results of the upper continental crust (UCC) from [51], as shown in Fig 10A. The graph shows that SiO_2_ content is close to that of the (UCC; TiO_2_, Al_2_O_3_, Fe_2_O_3_, MgO, CaO, Na_2_O, K_2_O, and P_2_O_5_ content tended to be lower than that in the UCC, but in some samples, MnO and CaO values were higher than that in the UCC. The study of the lithology and petrography showed that the samples had a calcareous cement binder, resulting in high CaO and MnO content of the samples, consistent with the results of the chemical analysis.

Trace elements and REEs in the rock samples were compared with those in the UCC from [52], and the result is presented in Fig 10B. The graph shows that the amount of Sc, V, Cr, Cu, Ni, and Zn tended to be lower than that in the UCC, indicating that the rock sample contained very few mafic minerals, which are found in small quantities when the rock is high in SiO_2_, excluding some calcrete samples (KK3-1) presenting high Cu (wt%). In addition, the lower Sr content than that in the UCC was owing to the high CaO content of the rock samples. Moreover, some sandy mudrock (KK7-2) presented high Ba content, suggesting that the rock sample contained a very large amount of potassium-bearing minerals (alkaline feldspars, mica, and clays). From the graph in Fig 10, it can be seen that only rock samples from the SKF (KK3 and KK6-8) have high Zr, Hf, and Y content, suggesting that these rocks may have a high zircon proportion in rock fragments, which is consistent with the petrographic classification of the rocks as lithic wacke, lithic arenite, and feldspathic lithic arenite. Furthermore, the provenance discrimination diagram from the major and minor oxide data (TiO_2_-Al_2_O_3_-Fe_2_O_3_-MgO-CaO-Na_2_O-K_2_O) [42] suggested that the PWF, SKF, PPF, and KKF originated from quartzose sedimentary rocks (Fig 7D).

Numerous studies have shown that the chemical composition of clastic rocks is partly influenced and controlled by tectonic settings, with different tectonic landscapes exhibiting different geochemical characteristics [41–45]. There are three main types of tectonic margins: passive continental margin, active continental margin, and oceanic island arc; these were classified using major and minor oxides in Fig 7A‒7C. These tectonic classifications [41–45] revealed that most are deposited in the passive continental margin or intracratonic basin. Furthermore, REE tectonic classification diagrams [41] demonstrate the samples were deposited in either a passive continental margin or intracratonic basin. Rare Earth Elements (REEs) are not mobile during weathering so are more reliable than major elemental analysis. Therefore, it was concluded that most rock samples are associated with passive continental margins or intracratonic basins (Fig 8).

**Fig 11:**
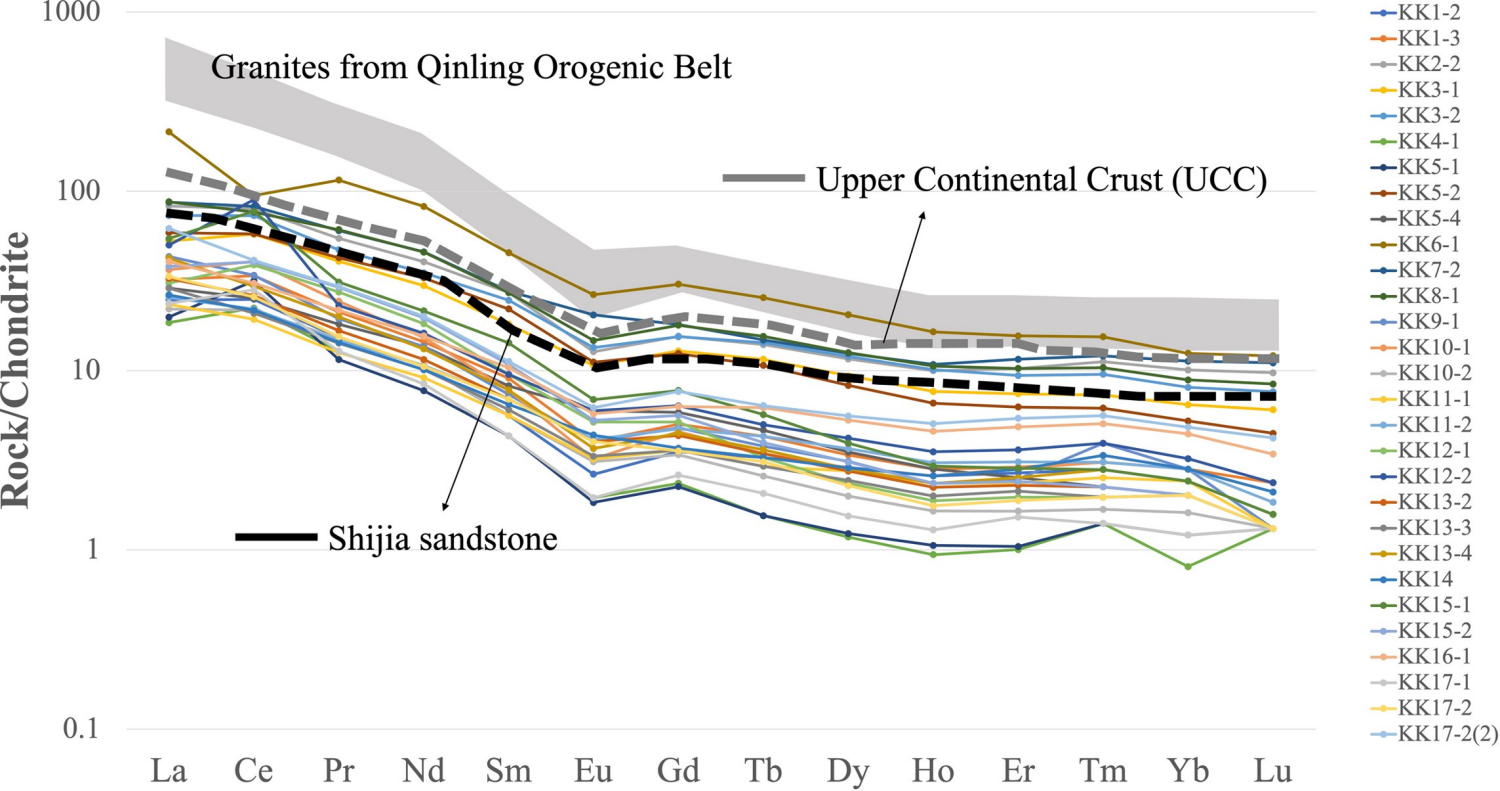
Chondrite-normalized REE patterns of the KKGp sedimentary rocks, Shijia sandstone, granites from Qinling Orogenic Belt, and UCC. Data for Shijia sandstone, granites from Qinling Orogenic Belt, UCC, and chondrite are from [51–54].

When comparing REEs in chondrite from [52, 53] concluded that the sandstones in the study area originated from quartzose sedimentary rocks deposited in the passive continental margin. REE patterns from Shijia sandstone [53] and REE patterns (Khorat Group) from this research were found to be similar (Fig 11) and their patterns also according to felsic igneous rocks from Qinling Orogenic Belt [54]. Therefore, it was concluded that the studied rock samples had similar origins and tectonic landscapes to those in the previously mentioned research. Although the rock samples from the two studies were collected at very different ages, the REEs within the rocks were not lost. Thus, the use of REEs is quite accurate and it can be concluded that studies of provenances and tectonic settings using rare earth data have some degree of reliability.

## Discussion

The KKGp comprises Mesozoic sedimentary rocks: sandstone, siltstone, mudstone, interbedded limestone lenses, calcrete, an evaporitic layer, and fossils. Many researchers have studied the sedimentology, stratigraphy, and paleocurrents of the formation [55–57]. Several studies attest to varied origins and tectonic environments [14, 15, 19, 21–27, 30, 41–43, 47].

**Fig 12:**
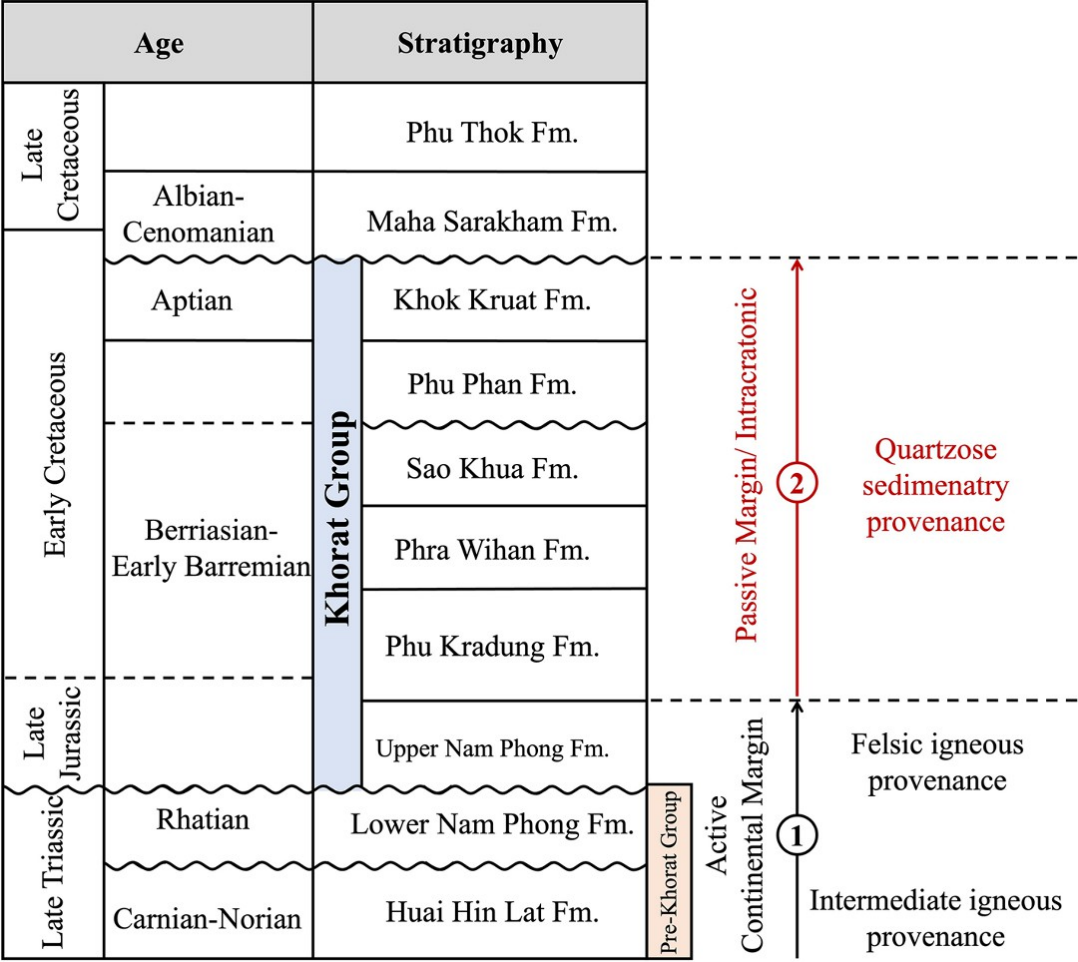
Simplified scheme of Khorat Group stratigraphy and tectonic setting from the late Triassic to early Cretaceous. Stratigraphic data from [12, 61].

Previous studies [10, 58, 59] have suggested that the late Triassic Huai Hin Lat and Nam Phong Formations originate from intermediate to felsic igneous rocks and quartzose sedimentary rocks and accumulate in the active continental margin to the passive continental margin or intracratonic basin (Fig 12). Based on lithology, petrography, and geochemistry, it has been suggested that the Jurassic to Cretaceous Phu Kradueng Formation, PWF, SKF, PPF, and KKF [60] originate from quartzose sedimentary rocks and accumulate in the passive continental margin or intracratonic basin (Fig 12).

**Fig 13:**
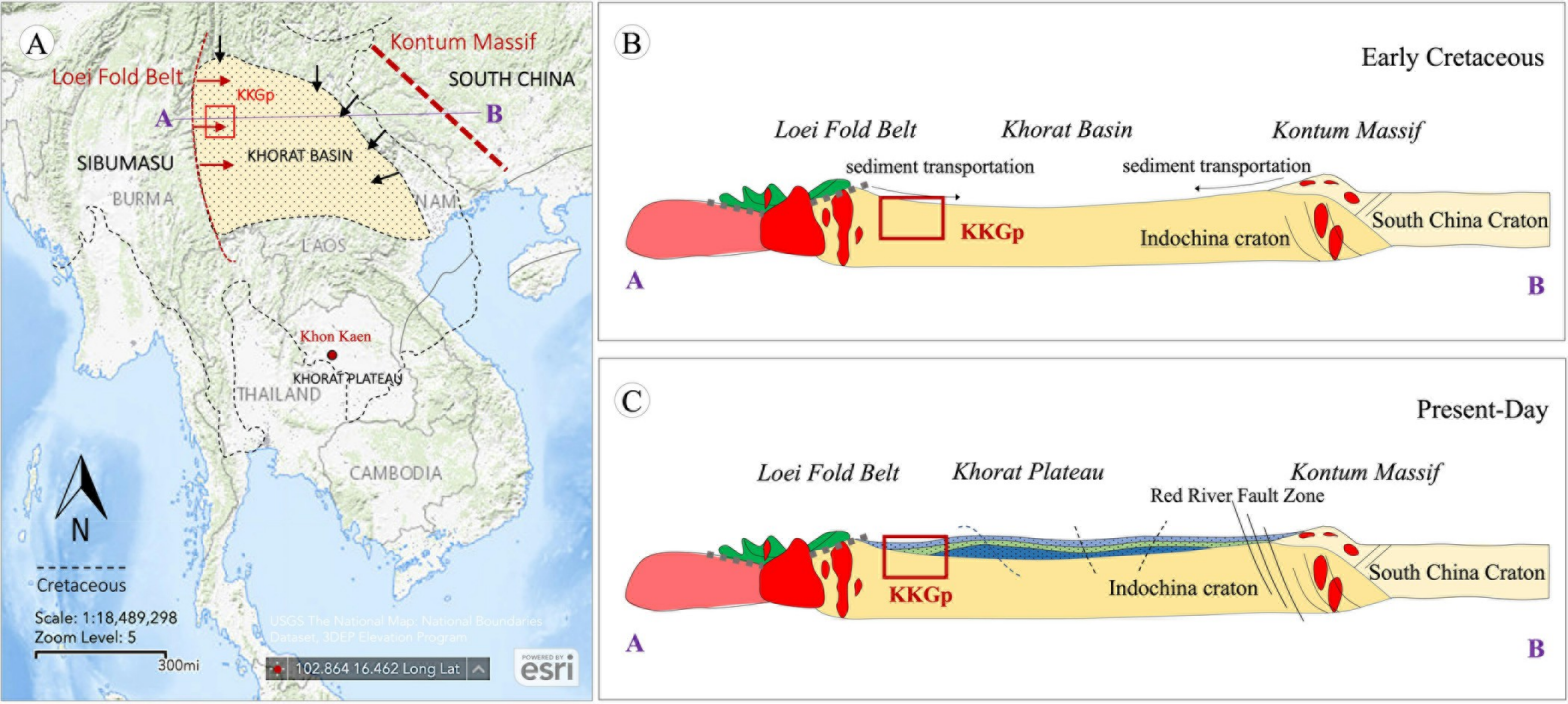
(A) a simplified map of the Khorat sediments and other basins containing Cretaceous recycled orogen sediments, (B) a tectonic model of the Khorat basin during the Early Cretaceous, and (C) present-day tectonics of the Khorat Plateau. The synthesis of the geotectonic evolution of Thailand is from [86, 87]. The location of the Khorat Basin and transportation (black arrow) of sediments are modified from [32, 70].

The Huai Hin Lat deposition in the sedimentary basin was created by the late Paleozoic collision of the microcontinents (Fig 13A) at a higher latitude than the present location or near Southeast Tibet [62]. Therefore, intermediate and felsic igneous rocks, as well as quartzose sedimentary rocks, were transported from the western part and accumulated in the basin [63]. Meanwhile, U-Pb geochronology and Lu-Hf isotopic signatures of the Huai Hin Lat formation (HHF) suggest that the western margin of the Khorat Plateau may have been part of an early Paleozoic arc system [64–70], which is consistent with the prominence of early Paleozoic zircons, or approximately 452 Ma [71, 72], and similar to the South China Terrane [73–76]. In addition, the 290 Ma zircon isotopic signature in the HHF is consistent with crystallization ages from granitoids within the Indochina Terrane that span from 310 to 203 Ma [54, 77–84] and is more evolved than the Khao Khwang Fold-Thrust Belt [85, 86]. Therefore, these ages may be associated with Late Carboniferous to Middle Permian subduction-related volcanic arcs as a result of the early stages of the South China collision with Vietnam and Indochina [73, 76], or the similarly timed Indosinian orogeny caused by the collision of the Sibumasu and Indochina Terranes [58, 83].

Over time, these two microcontinents became more stable, and the Nam Phong Formation occurred (Fig 13B) from sediments of felsic igneous rocks and quartzose sedimentary rocks [59], bounded below and above by major unconformities, deposited in the NW-SE trending basin in similarity to south Vietnam and NE Cambodia [84]. The ostracod assemblage and microfacies in the lower Jurassic Nam Phong Formation, above the oldest known dinosaur footprints site in Thailand, indicate deposition in a shallow and low-energy fluvial-lacustrine regime [85]. During this period, a contractional fold belt in the basin developed during the Early and Middle Jurassic affected by the Jurassic tectonic of the southeastern Indochina Terrane [84].

Many researchers suggest that the Khorat Group was essentially deposited in a layer-cake fashion, accompanied by local faulting [58, 88], whereas Racey described the Khorat Group as being deposited in a craton interior setting or intracontinental basin [12]. In addition, stratigraphic investigation described that the Khorat Group in the Lom Kao (the western part) and Phu Phan Mountain (the eastern part) as being generally oriented in a westerly direction from the South China Terrane [63]. Considering the orientations of the cross-bedding data in the field, the paleocurrent pattern in the PWF and PPF was in a westerly direction [32].

Collisional orogens are one of the factors that affect the sedimentary rift basin of the tectonic passive continental margins [39, 89]. This event may apply to the Khorat Basin, which was developed as a series of major half-grabens in the extensional event during the Triassic (Fig 13C). In addition, the Indosinian orogeny during the early Cretaceous may have caused the collapse of the over-thickened crust, and multi-sequence continental sediments were deposited during the Jurassic and Cretaceous [90]. Usually, the intracontinental basin is flanked by mountains on both sides. The drainage transports sediments to the basin on each side until it accumulates as a stratum. Moreover, dinosaur footprints (small theropods *Carmelopodus* isp.) were discovered in the PWF [16, 91, 92], supporting the premise that dinosaurs lived in this terrane during the Jurassic-Cretaceous in a braided stream environment [30].

U-Pb zircon dating of sediments in the Khorat Group were reported as 2,456±4 Ma, 2,001±4 Ma, 251±3 Ma, and 168±2 Ma [1], whereas Wang et al. identified them as 2,500 Ma and 1,860 Ma [93]. These ages are similar to the sediments in the Kangdian basement, Sichuan, Songpan-Garze, Qiangtang, Yidun geological domains, and Qinling Orogen [40, 54, 74–81] potentially representing the provenances.

The thick sequence of SKF continental redbeds in KKGp is mostly discovered to be abundant and diverse dinosaur fossils, especially the first Thai dinosaur fossil “*Phuwiangosaurus sirindhornae*” [14, 23, 94]. In addition, the SKF first found four other dinosaur species: *Kinnareemimus khonkaenensis*, *Siamotyrannus isanensis*, *Siamosaurus suteethorni*, and *Phuwiangvenator yaemniyomi* [14, 20, 21, 24, 25], as well as Jurassic to mid-Cretaceous vertebrate and Mollusca fossils [11] deposited in the semi-arid meandering rivers and swamps along the riverbank [30, 31]. According to the radiometric dating of detrital zircons, the SKF was deposited no later than 133.8 Ma. [95]. Similarly, the detrital zircon age pattern of the early Cretaceous in Southeast Asia closely resembles that of the Qinling-Dabie and Songpan-Ganzi orogenic belts or South China Volcanic Arc [95–97].

On the other hand, the 470–430 Ma zircon isotopic characterization was also found in the late Cretaceous–early Paleogene sandstones (Phu Khat Formation) further northwest of the Khorat Plateau [1, 98]. This evidence suggests the western Indochina Terrane may also be related to the Proto-Tethys closure in the early Paleozoic (500–420 Ma) [64–70].

Based on the age constraints of detrital zircons in the Khorat Plateau (PWF-PPF), the sedimentary rocks were from a major early Cretaceous drainage disruption across Southeast Tibet [60]. According to Jurassic-early Cretaceous zircons (U-Pb dating and U-Pb-Hf compositions) from the Khorat Plateau [60], the sedimentary rocks of the PWF, SKF, PPF, and KKF were largely recycled from multiple source terranes, with some possibly transported from the Sibumasu terrane or terrane in the western flank (Fig 13A), which is consistent with our petrographic and chemical results. According to the cross-section illustrated in Fig 1, the KKGp shows the fold structures (Fig 13B) that may be affected by the extensive Palaeogene deformation and neotectonics (Miocene–Pleistocene) during the Himalayan orogeny event [99]. The fold and strike-slip fault structures along the NW–SE direction in the Khorat Plateau represent the dominant tectonic deformation fabric evolved by compression [100]. The regional structures and tectonic evolution were recorded by fold structures in the KKGp, supporting that tectonically this region is located on the western margin of the composite Indochina Terrane and deposited in diverse tectonic settings.

## Conclusion

The geological record of the KKGp involves Mesozoic sandstone of the Khorat Group, including the PWF, SKF, PPF, and KKF, representing the western part of the Khorat Plateau. The SKF is the main stratum for fossil exploration, especially dinosaur footprints and dinosaur bones. According to field observation, lithology, petrography, and geochemistry, the conclusions and interpretations regarding the sedimentary rocks of the KKGp are as follows:

- Field investigation and lithological data suggest that Mesozoic sandstones of the Khorat Group have several sequences of maroon and white clastic sedimentary successions, which consist mainly of sandstone, pebbly sandstone, and siltstone interbedded with calcrete, conglomerate, and some evaporite layers.
- Petrographical data (Qt–F–Lt) and geochemical data (SiO_2_, Al_2_O_3_, Na_2_O, K_2_O, and Fe_2_O_3_) are consistent; the PWF is made up of quartz arenite, litharenite, sublitharenite, and subarkose. Moreover, the SKF comprises subarkose and sublitharenite. Furthermore, the PPF comprises quartz arenite, sublitharenite, and arkose, similar to the KKF.
- Using Qm, Qp, F, Lv, Lm, and Ls, petrographic data classified the provenance from diverse tectonics, including recycled orogen with quartzose recycled, craton interior, mixed sources, subduction complex, and collision suture fold-thrust.
- Major oxide relationships (TiO_2_, Fe_2_O_3_, MgO, K_2_O, Na_2_O, SiO_2_, and Al_2_O_3_) identified that the sources of the Mesozoic sandstones were quartzose sedimentary rocks of the passive margin, and some were felsic-intermediate igneous rocks of the active continental arc.
- Sandstones of the KKGp were from mixed provenances (quartzose sedimentary and felsic-intermediate igneous rocks) related to the UCC, characterized by La, Yb, Ce, Th, Hf, Sc, Zr, and chondrite-normalized REE patterns.
- The sandstones in the KKGp were predominantly sourced from the exposed proximal rocks in the terrane, from the northwestern recycled orogen, based on their maturity, grain characteristics, and previous U-Pb Lu-Hf isotopic signatures.
- This publicity could increase the international scientific value of KKGp allowing it to achieve the status of a UNESCO Global Geopark and facilitate development of sustainable geotourism. However, the results of this study based on the relatively small dataset and geochemistry remain inconclusive. Thus, additional electron probe microanalyses (EPMA) for heavy metal minerals and isotopes are required for additional resolution.

## Supporting information

S1 Table Lithological data of sedimentary rocks in Khon Kaen Geopark. (XLSX)

S2 Table Petrographical data of sedimentary rocks in Khon Kaen Geopark. (XLSX)

S3 Table Major oxides of sedimentary rocks in Khon Kaen Geopark by XRF analysis. (XLSX)

S4 Table Trace and rare earth elements of sedimentary rocks in Khon Kaen Geopark by ICP analysis. (XLS)
